# Structure of native HIV-1 cores and their interactions with IP6 and CypA

**DOI:** 10.1126/sciadv.abj5715

**Published:** 2021-11-19

**Authors:** Tao Ni, Yanan Zhu, Zhengyi Yang, Chaoyi Xu, Yuriy Chaban, Tanya Nesterova, Jiying Ning, Till Böcking, Michael W. Parker, Christina Monnie, Jinwoo Ahn, Juan R. Perilla, Peijun Zhang

**Affiliations:** 1 Division of Structural Biology, Wellcome Trust Centre for Human Genetics, University of Oxford, Oxford OX3 7BN, UK.; 2 Electron Bio-Imaging Centre, Diamond Light Source, Harwell Science and Innovation Campus, Didcot OX11 0DE, UK.; 3 Department of Chemistry and Biochemistry, University of Delaware, Newark, DE, USA.; 4 Department of Structural Biology, University of Pittsburgh School of Medicine, Pittsburgh, PA, USA.; 5 EMBL Australia Node in Single Molecule Science and ARC Centre of Excellence in Advanced Molecular Imaging, School of Medical Sciences, UNSW, Sydney, Australia.; 6 Department of Biochemistry and Molecular Biology, Bio21 Molecular Science and Biotechnology Institute, The University of Melbourne, Parkville, Victoria 3010, Australia.; 7 St. Vincent’s Institute of Medical Research, Fitzroy, Victoria 3065, Australia.; 8 Chinese Academy of Medical Sciences Oxford Institute, University of Oxford, Oxford OX3 7BN, UK.

## Abstract

The viral capsid plays essential roles in HIV replication and is a major platform engaging host factors. To overcome challenges in study native capsid structure, we used the perfringolysin O to perforate the membrane of HIV-1 particles, thus allowing host proteins and small molecules to access the native capsid while improving cryo–electron microscopy image quality. Using cryo–electron tomography and subtomogram averaging, we determined the structures of native capsomers in the presence and absence of inositol hexakisphosphate (IP6) and cyclophilin A and constructed an all-atom model of a complete HIV-1 capsid. Our structures reveal two IP6 binding sites and modes of cyclophilin A interactions. Free energy calculations substantiate the two binding sites at R18 and K25 and further show a prohibitive energy barrier for IP6 to pass through the pentamer. Our results demonstrate that perfringolysin O perforation is a valuable tool for structural analyses of enveloped virus capsids and interactions with host cell factors.

## Teaser

High-resolution cryoET structures of native HIV-1 capsid in complex with IP6 and CypA are enabled by viral membrane perforation.

## INTRODUCTION

Retroviruses are enveloped single-stranded positive-sense RNA viruses. Upon infection, the RNA genome is reverse-transcribed into a double-stranded DNA, which is then integrated into the host genome. The retroviral capsid is a container that encapsulates the viral genome and protects it from host innate immune responses during the early stage of infection. It is also a major platform that engages many host dependency and restriction factors via higher-order surface pattern recognition. The HIV-1 capsid is made of capsid protein (CA), which contains two separate domains, CA N-terminal domain (CA_NTD_) and CA C-terminal domain (CA_CTD_), connected by a flexible linker. The capsid is organized as a fullerene-like cone, within which about 200 CA hexamers and 12 pentamers make up the conical lattice (*1*–*3*). The capsid plays multiple roles during the early stage of HIV-1 replication, including protecting the genome from cellular innate immune responses and fostering reverse transcription, as well as regulating intracellular transport and entry into the nucleus (*4*, *5*). Many of these functions are affected by its interactions with host cell factors (*4*, *5*).

The breaking or disassembly of the HIV-1 capsid, termed “uncoating,” is a critical process during retroviral replication. Mutations in CA or interactions with specific host factors, such as cyclophilin A (CypA), tripartite motif–containing protein 5α (TRIM5α) and TrimCyp, cleavage and polyadenylation specificity factor 6, and the human myxovirus resistance 2 (Mx2/MxB), can alter capsid stability and affect uncoating (*4*). In addition, small molecules, including cellular metabolite inositol hexakisphosphate (IP6) (*6*–*8*), PF74 (*9*, *10*), GS-CA1 (*11*), and GS-6207 (*12*, *13*), have been shown to modulate capsid assembly and stability. Host restriction factors, such as TRIM5α, TrimCyp, and MxB, recognize the capsid lattice pattern, rather than individual capsomeres, and preferentially bind the assembled capsid (*14*–*16*). However, because of the metastable property of HIV-1 capsid (*17*), isolating fully intact native cores in quantities and concentrations suitable for high-resolution structural analyses has been challenging. An additional challenge is posed by the morphological heterogeneity of retroviral capsids. Hence, in vitro–assembled capsid tubes, which generally require high salt for assembly and stability, have served as a surrogate to study capsid assembly and its interactions with host cell factors over the past two decades (*1*, *18*–*22*). Structures of curved tubular capsid assemblies analyzed by cryo–electron microscopy (cryoEM) (*1*, *19*, *21*, *23*) were recently determined to near-atomic resolution with and without CypA (*18*, *19*) but with the caveat of high salt (1 M) that could affect other host factor binding. Only recently were structures of CA capsomere hexamers and pentamers in membrane-enclosed virus particles determined, at 6.8- and 8.8-Å resolution, respectively, by cryo–electron tomography (cryoET) and subtomogram averaging (STA) (*24*). However, these membrane-enclosed particles do not allow access of host factors or small molecules to viral capsid inside.

To overcome these challenges, we devised a strategy to directly image authentic HIV-1 cores, in the absence or presence of exogenous host factors and small molecules, by treating virus particles with perfringolysin O (PFO). PFO is a cholesterol-dependent cytolysin secreted by the pathogenic *Clostridium perfringens* that forms giant homo-oligomeric pores with diameter ~40 nm on cholesterol-rich membrane bilayers (*25*), such as membranes of HIV-1 particles. PFO treatment makes the core accessible to exogenous factors (*26*) without removing the core from the virus-like particles (VLPs) or compromising its stability because of the purification or isolation procedure. We imaged PFO-perforated HIV-1 VLPs by cryoET STA and determined the structures of HIV-1 native capsomeres in the apo state and in the presence of IP6. Previous studies established that IP6 binds to the central R18 pore, stabilizing the CA hexamer (*7*, *8*, *27*–*29*) and promoting endogenous reverse transcription (*28*). We found that IP6 binds native capsid at the sites both above and below the CA R18 ring, distinct from the previous result from native virions (*7*, *24*) but consistent with the crystal structures of cross-linked CA hexamers in complex with IP6 or IP5 (*6*, *8*). Free energy calculations (*12*, *27*) of capsid interacting with IP6 support the two binding sites at R18 and K25 and showed different profiles between hexamer and pentamer.

We previously demonstrated that host dependency factor CypA binds CA tubular assemblies and bridges the capsomeres of CA lattices and stabilizes the capsid via two additional noncanonical binding sites (*18*, *19*). To investigate the interactions between CypA and native conical capsid, we analyzed PFO-treated HIV-1 VLPs in the presence of CypA or a tetrameric fusion protein CypA-DsRed, which was developed as an efficient capsid label for live-cell imaging assays (*30*), and in the presence of IP6 as expected in the cytoplasm. While it is still challenging to resolve CypA (18 kDa) in cryoET, our results demonstrate that the avidity of tetrameric CypA-DsRed (~200 kDa) toward capsid is achieved through simultaneous binding of two CypA molecules to the capsid. On the basis of the high-resolution cryoET structure of native capsid, we further derived a realistic all-atom model of the complete capsid comprising 241 hexamers and 12 pentamers by molecular dynamics (MD) simulations. Overall, this work demonstrates that PFO perforation of viral membranes is a valuable tool for structural analysis of the native capsid and interactions with host cell factors. This approach can broadly apply to study other enveloped viruses in their native states.

## RESULTS

### PFO-perforated HIV-1 VLPs for in situ structural analysis

**Fig. 1.**
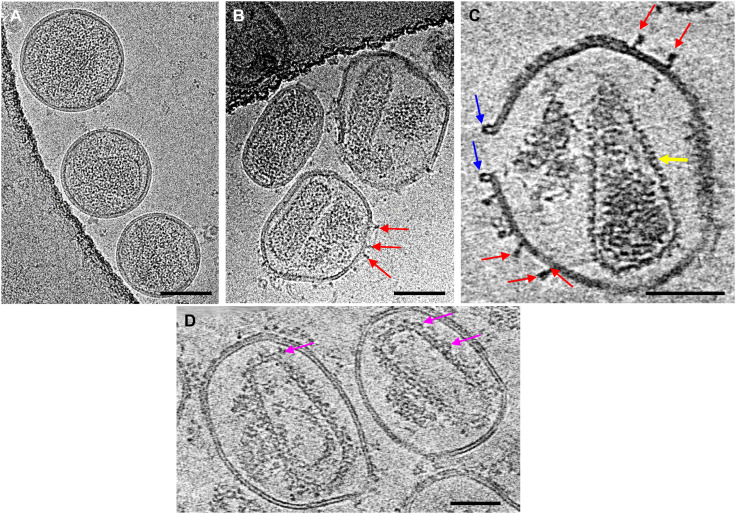
CryoEM of PFO-perforated mature HIV-1 VLPs. (**A** and **B**) CryoEM projection images of *Env^−^* HIV-1 mature VLPs without (A) and with (B) PFO treatment. (**C** and **D**) Tomographic slices (6.3 nm thick) of PFO-treated *Env^−^* HIV-1 mature VLPs in the absence (C) and in the presence of IP6 and CypA-DsRed (D). Arrows point to PFO protein in the prepore (red) and pore conformation (blue), the mature CA lattice (yellow), and CypA-DsRed density (pink). Scale bars, 100 nm (A and B) and 50 nm (C and D).

To circumvent the difficulties of working with delicate HIV-1 cores, we used a pore-forming protein, PFO, to puncture a limited number of ~30- to 40-nm holes into the viral membrane while otherwise preserving membrane integrity and its ability to contain the capsid (Fig. 1), i.e., without completely solubilizing and removing the lipid bilayer as a typical HIV-1 core prep. For biosafety purposes, we worked with noninfectious VLPs that were produced by transfecting cells with plasmid DNA that expressed HIV-1 Gag structural proteins as well as viral replication enzymes. The PFO concentration and incubation time were optimized to yield just a few pores, typically two to four, per VLP on average, as assessed by negative staining EM and cryoET (Fig. 1). The PFO molecules bound to virus membranes in both prepore (Fig. 1C, red arrows) (*25*) and pore conformations (Fig. 1C, blue arrow) (*25*). The appearance of PFO in prepore and pore is consistent with a collapse of 30 Å toward the membrane in the prepore to pore transition (*31*). During HIV-1 maturation, upon viral protease cleavage of Gag polyprotein, approximately half of the 2500 CA molecules released by Gag cleavage remain soluble, whereas the other half assemble into the mature conical-shaped capsid (*32*–*34*). PFO perforation effectively released the free CA protein through PFO pores, substantially enhancing the signal-to-noise ratio in cryoEM images and tomographic slices (Fig. 1, B and C), compared to untreated particles (Fig. 1A). PFO pores allowed small molecules and host cell factors access to the native viral capsid (*26*). Incubation of PFO-perforated VLPs with CypA or CypA-DsRed resulted in additional densities decorating the core surface, presumably from CypA or CypA-DsRed (Fig. 1D, magenta arrows), thus allowing structural analysis of the native core in complex with host factors.

### Structures of native CA hexamer and pentamer and an all-atom realistic capsid model

**Fig. 2.**
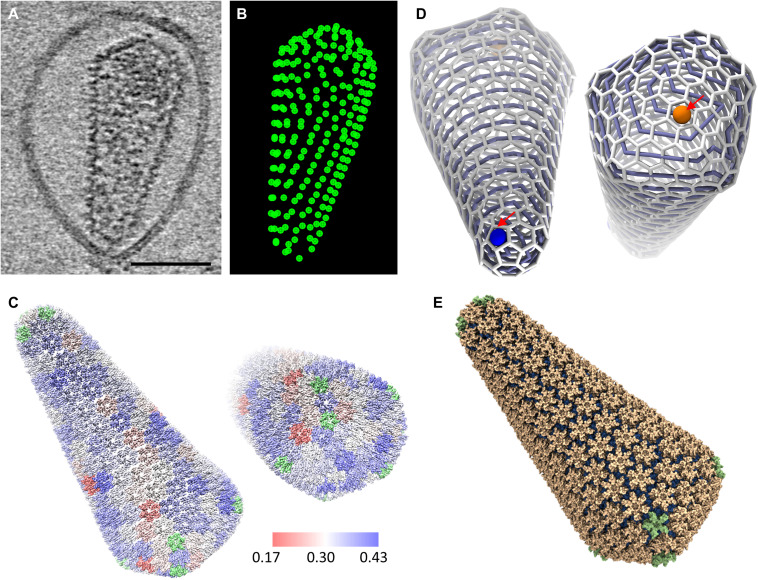
Reconstruction of a complete native HIV-1 mature capsid comprising 241 hexamers and 12 pentamers. (**A**) A tomographic slice (6.3 nm thick) of a PFO-treated *Env^−^* HIV-1 mature VLPs. Scale bar, 50 nm. (**B**) A bead model showing the coordinates of hexamers from emClarity template matching. (**C**) The reconstructed density map of a complete native HIV-1 mature capsid, with refined positions and maps of hexamers (5.8-Å resolution) and pentamers (8.6-Å resolution). Hexamers are colored according to their cross-correlation values between the raw data and the subtomogram-averaged map (range, 0.17 to 0.43 from red to blue). The pentamers are colored in green. (**D**) Trivalent network representation of the fullerenic model of the native HIV-1 capsid based on the reconstruction in (C). Starting from a pentamer at the tip (blue) and finishing at a hexamer at the base (orange), a self-avoiding spiral can connect all hexamers and pentamers in the HIV-1 core. We encounter the ring spiral pentamer positions at 1, 2, 9, 15, 17, 198, 203, 207, 212, 217, 230, and 239. (**E**) Molecular surface of the atomistic MD model guided by the reconstructed density map of a complete capsid shown in (C). Scale bars, 100 nm (A and B) and 50 nm (C and D).

**Fig. 3.**
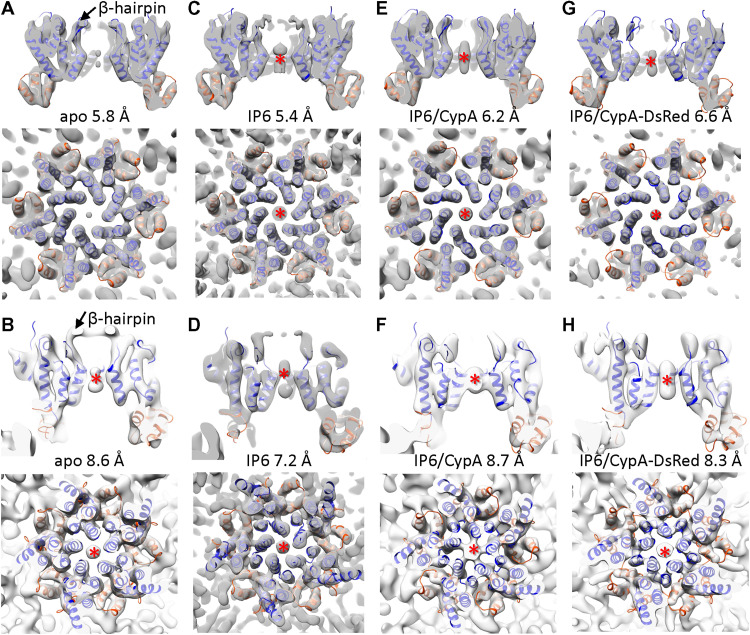
Structures CA hexamers and pentamers from native cores. (**A** and **B**) Subtomogram averages of CA hexamer (A) and pentamer (B) from native PFO-treated HIV-1 VLPs (apo state). (**C** and **D**) Subtomogram averages of CA hexamer (C) and pentamer (D) from PFO-treated VLPs in the presence of IP6. (**E** and **F**) Subtomogram averages of CA hexamer (E) and pentamer (F) from PFO-treated VLPs in the presence of IP6 and CypA. (**G** and **H**) Subtomogram averages of CA hexamer (G) and pentamer (H) from PFO-treated VLPs in the presence of IP6 and CypA-DsRed. Density maps are shown in sectional views from side and top, superimposed with the CA atomic model (PDB 4XFX), with CA_NTD_ and CA_CTD_ fitted separately, colored in blue and orange, respectively. The resolution of each map is indicated. Red asterisks indicate the IP6 density in CA hexamers and pentamers.

We first analyzed CA hexamer and pentamer structures in wild-type (WT) cores inside of PFO-perforated VLPs using cryoET STA with emClarity (Table 1) (*35*). The hexamers and pentamers on the capsid were identified by template matching (Fig. 2, A and B), with manual inspections considering the local geometry. We obtained subtomogram-averaged maps of CA hexamer and CA pentamer at resolutions of 5.8 and 8.6 Å, respectively (Fig. 3, A and B, and fig. S1), into which individual CA_NTD_ and CA_CTD_ structures [Protein Data Bank (PDB) 4XFX] were rigid body–fitted (Fig. 3, A and B). Intriguingly, we observed very weak central density for IP6 in the hexamer subtomogram average (Fig. 3A), in contrast to the hexamer subtomogram average from membrane-enclosed virions (*24*). This difference cannot be attributed to the map quality as the new map is, in fact, at a slightly higher resolution with less than a half of subtomograms (table S1) owing to improved image contrast with PFO treatment. A likely scenario is that IP6 molecules, initially packaged inside VLPs (estimate ~300 to 400 IP6 per particle if each Gag hexamer binds one IP6) (*6*), were released from the VLPs through PFO pores when diluted into IP6-free buffer. The central density of IP6 was stronger in the pentamer than hexamer when contoured at the same level (Fig. 3B, red asterisk), suggesting that IP6 binds with higher affinity to pentamers than hexamers.

**Table 1.** CryoET data collection of perforated mature VLPs and STA statistics of CA hexamers and pentamers.

**Samples**	**HIV-1**	**HIV-1 + IP6**	**HIV-1 + IP6 + CypA**	**HIV-1 + IP6 + CypA-DsRed**
**Acquisition settings**				
Microscope	FEI Titan Krios	FEI Titan Krios	FEI Titan Krios	FEI Titan Krios
Voltage (keV)	300	300	300	300
Detector	Gatan Quantum K2	Gatan Quantum K3	Gatan Quantum K2	Gatan Quantum K2
Energy filter	Yes	Yes	Yes	Yes
Slit width (eV)	20	20	20	20
Super-resolution mode	No	No	No	No
Å per pixel	1.043	1.06	1.048	1.048
Defocus range (μm)	−2 to −3	−2.5 to −7	−1.5 to −3	−1.5 to −3
Defocus increment (μm)	0.3	0.3	0.3	0.3
Acquisition scheme	−60/60, 3° step, group 3	−60/60, 3° step, group 3	−60/60, 3° step, group 3	−60/60, 3° step, group 3
Total dose (electrons/Å^2^)	120	120	120	120
Number of frames	10	10	10	10
Number of tomograms	101	109	106	121
**Structure determination**				
Cores	157	481	218	282
No. of hexamers/pentamers	32,114/901	82,837/4506	42,255/1186	49,689/622
Resolution (Å) of hexamers/ pentamers	5.8/8.6	5.4/7.2	6.2/8.7	6.6/8.3
B-factor–applied hexamers/ pentamers	−100/−10	−100/−10	−100/−10	−100/−10
Data-deposited hexamers/ pentamers	EMD-12452/EMD-12456	EMD-13423/EMD-13422	EMD-12454/EMD-12457	EMD-12455/EMD-12458

The CA hexamer shows clear density of CA_NTD_ and CA_CTD_ helices, with a linker density connecting the domains. The CA_CTD_ dimer interface is well defined. The pentamer map, although at lower resolution due to the limited number of subtomograms available, confirmed structural features present in the intact virion at a similar resolution (*24*), which differ from the crystal structure of in vitro cross-linked pentamers (*36*). We refined the position and orientation of each hexamer and pentamer within a representative VLP shown in Fig. 2 (A and B) and reconstructed the density map of a complete native HIV-1 capsid (Fig. 2C). The reconstructed HIV-1 capsid comprises 241 hexamers and 12 pentamers at 5.8- and 8.6-Å resolution, respectively. On the basis of the coordinates and orientation of the capsomeres in the capsid, we constructed a geometry-constrained fullerenic model (Fig. 2D) (*21*) and, using the structures of the capsomeres, built an atomic model of a complete capsid by all-atom MD simulations (Fig. 2E). There is very little deviation between the capsomere positions of experimentally refined (Fig. 2C) and geometry constrained (Fig. 2E) [root mean square deviation (RMSD) = 0.95], indicating that this is an accurate and realistic atomistic capsid model, thus a considerable improvement over the previous theoretical model (*21*).

### Structures of CA capsomeres in the presence of IP6, IP6/CypA, and IP6/CypA-DsRed

**Fig. 4.**
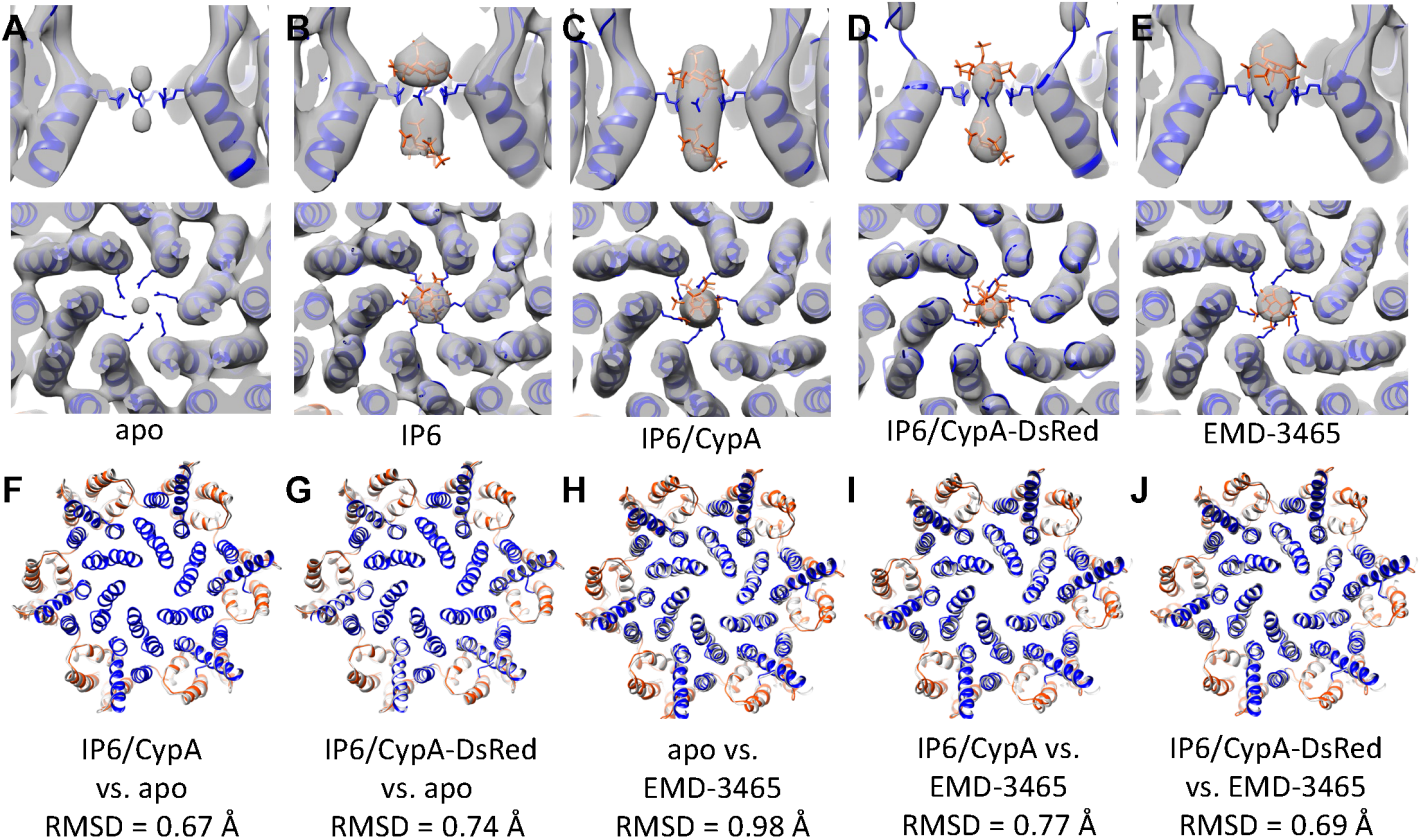
Comparison of CA hexamer structures with/without IP6. (**A** to **E**) Comparison of IP6 density in apo (A), IP6 complex (B), IP6/CypA complex (C), IP6/CypA-DsRed complex (D), and EMD-3465 (E). R18 side chain is shown in blue. Two IP6 molecules (red) were modeled in by aligning the CA hexamer to the cross-linked CA hexamer in IP6 cocrystals (PDB 6BHT). (**F** and **G**) Comparison of CA hexamer backbone between apo (colored gray) and IP6/CypA complex (F) and IP6/CypA-DsRed complex (G). (**H** to **J**) Comparison of CA hexamer backbone between EMD3465 (colored gray) and apo (H), IP6/CypA complex (I), and IP6/CypA-DsRed complex (J).

Using the same strategy, we further determined structures of CA hexamers and pentamers from PFO-perforated VLPs in the presence of IP6, and IP6 together with CypA or CypA-DsRed at similar resolutions as in the apo state (Fig. 3, C to F, and fig. S1). Previous studies have shown that IP6 binds the mature HIV-1 capsid at the center of the CA hexamer coordinated by a ring of six R18 from CA_NTD_ (*6*, *7*). Comparing with the CA hexamer map in the apo state (Fig. 3A), we observed a central density coordinating the R18 ring in all the IP6, IP6/CypA, and IP6/CypA-DsRed hexamer maps (Fig. 3, B to D). This density is not an artifact of sixfold symmetrization or B-factor sharpening because it is also present in subtomogram averages without applying sixfold symmetry throughout the processing and is within a confidence map set at 1% false discovery rate threshold (fig. S2) (*37*). The IP6 density occupies an area both above and below the R18 ring in all hexamer maps in the presence of IP6 (Fig. 4, B to D) rather than the only above the R18 ring in membrane-enclosed virions (Fig. 4E). Two IP6 molecules were found bound at both above and below the R18 ring in crystal structures of cross-linked mature CA hexamer in complex with IP6 (PDB 6BHT) (*6*) and a recent MD simulation study (*29*). In all conditions (apo, IP6, IP6/CypA, and IP6/CypA-dsRed), the IP6 density was present in the center of the pentamer (Fig. 3, B, D, F, and H), similar to that in membraned virions (*24*).

A comparison of CA hexamer backbones in the presence and absence of IP6/CypA or IP6/CypA-DsRed indicates that there is essentially little deviation in these CA capsomere structures (Cα backbone RMSD = 0.75 and 0.55 Å; Fig. 4, E and F), demonstrating that IP6 and CypA binding have little impact on the overall structure of the CA capsomere. This is consistent with the previous study on in vitro–assembled CA tubes (*19*), which showed that the apo and CypA-bound CA tubes with the same helical symmetry had the same overall structure. The RMSD between the hexamers in PFO-treated and membraned HIV-1 capsid (EMD-3465) is also small (RMSD = 0.69 to 0.98 Å) (Fig. 4, G to I), suggesting minimal effect of PFO treatment on capsid structure.

### Free energy profiles along the central pore of native CA hexamers and pentamers

**Fig. 5.**
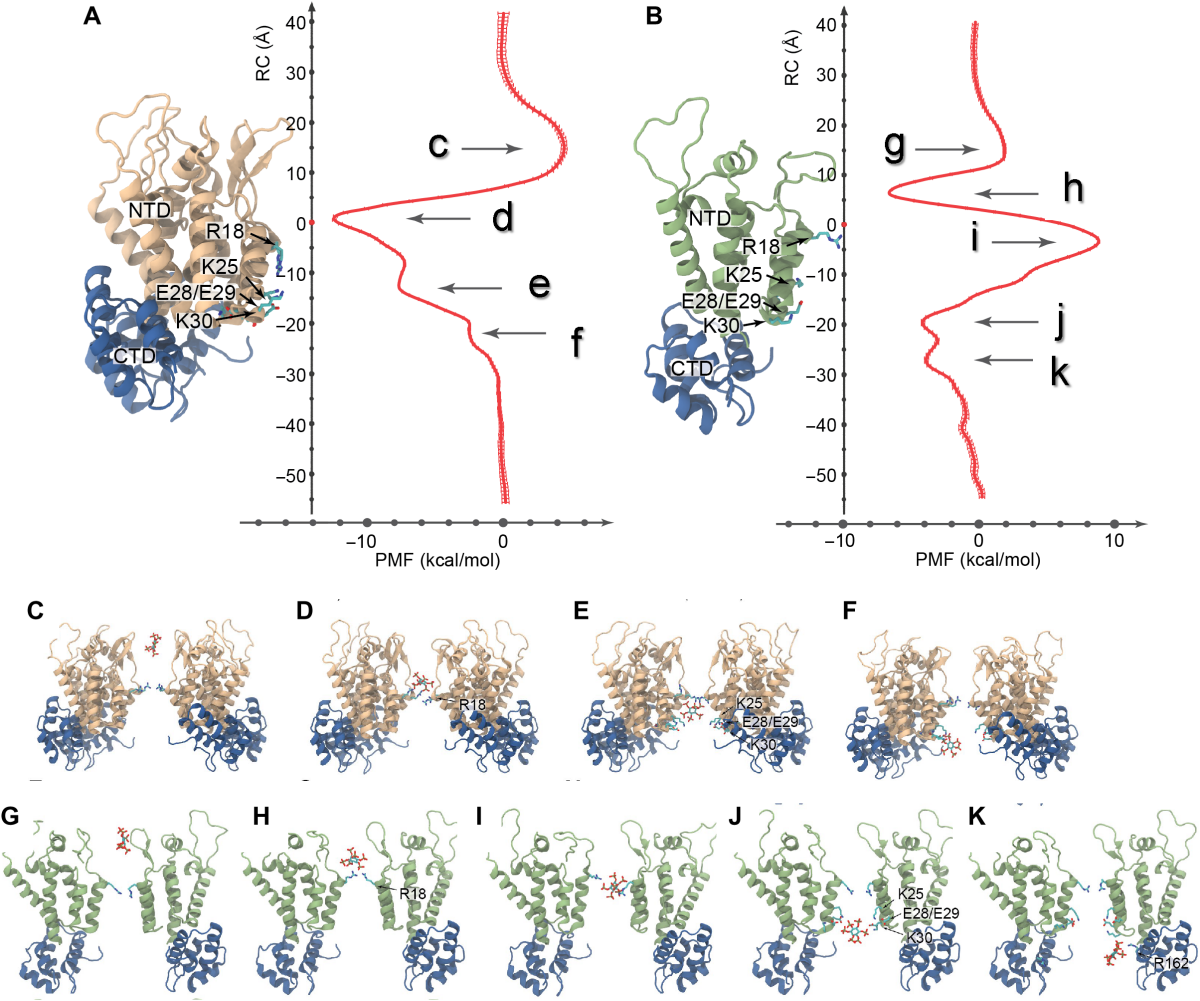
Free energy profiles along a reaction coordinate for the interactions between IP6 and native HIV-1 CA hexamers and pentamers. (**A**) PMF for an IP6 molecule interacting with the central pore of a hexamer (plotted with SE). A maximum (labeled c) and three minima (labeled d, e, and f) are observed in the PMF. (**B**) PMF for an IP6 molecule interacting with the central pore of a pentamer. Two maxima (labeled g and i) and three minima (labeled h, j, and k) are observed in the PMF. (**C** and **G**) Interactions between β-hairpins in hexamers/pentamers and IP6 result in unfavorable energetics. (**D** and **H**) When located in the hexamer/pentamer gauntlet, IP6 favorably interacts with the R18 ring. (**E**) The most favorable interaction of IP6 with hexamers is mediated by residues R18 and K25. (**F**) In hexamers, IP6 favorably interacts with R18 and K30, resulting in a third PMF minima. (**I**) IP6 interacts unfavorably when located in the constricted region of the pentamer surrounded by R18. (**J**) Favorable interactions between IP6 and pentamers occur when the former interacts with the KEEK motif in helix 1. (**K**) A third minima results from the interaction between IP6 with R162. RC, reaction coordinate.

Potential of mean force (PMF) profiles along the central cavity of native CA hexamers and pentamers were derived using Hamiltonian replica-exchange umbrella sampling simulations (*27*). MD flexible fitting was used to derive atomistic models usable in MD simulations of the native hexamer and pentamer (Fig. 5, A and B). The PMF profile for the native hexamer differed slightly from the previously derived PMF for flat hexamers and revealed three energy minima: one located on top of R18 and two below R18 (Δ*G* = −12 kcal/mol, Δ*G* = −8 kcal/mol, and Δ*G* = −2 kcal/mol, respectively). A small barrier (Δ*G* = +4 kcal/mol) was observed near the β-hairpin region (Fig. 5, A and C). Binding of IP6 below R18 is coordinated by residues K25 and K30, consistent with the IP6 positions observed in native cores by cryoET (Fig. 3, C and E). For native CA pentamers, three energy minima and a large energy barrier were observed. The energy barrier appears directly adjacent to the center of the R18 ring (Δ*G* = +9 kcal/mol; Fig. 5, B and I). The first minimum is located on top of R18, while two minima were observed below R18 (Δ*G* = −7 kcal/mol, Δ*G* = −4 kcal/mol, and Δ*G* = −4 kcal/mol, respectively). Binding of IP6 to the bottom minima of the native CA pentamer is coordinated by K25/E28/E29/K30 and by R162. The observation that K25 coordinates binding of IP6 to both hexamers and pentamers has been previously postulated (*27*) and supported by recent crystal structures of cross-linked CA hexamer with IP6 or IP5 (*6*, *8*). Nevertheless, direct observation by cryoET of second IP6 binding to native capsid coordinated by K25 confirms biochemical and infection experiments in which K25 substitutions resulted in impaired virus production and infectivity (*8*).

Overall, the PMFs revealed a probabilistic interpretation of the binding and unbinding of IP6 to native hexamers and pentamers. In general, IP6 can bind on either side of a hexamer and can translocate through the R18 ring with a small energetic penalty (Δ*G* = +4 kcal/mol). Nevertheless, once IP6 is bound, diffusion from the capsid presents a higher energetic barrier compared to diffusion into the capsid (Δ*G* = +12 kcal/mol); therefore, diffusion of IP6 from CA hexamers is more likely to occur into the capsid. We previously highlighted that IP6 may facilitate deoxynucleotide triphosphates (dNTPs) for inward passage through the hexamer pore, which is postulated to support reverse transcription (*27*, *38*). Pentamers are also able to bind IP6 above and below R18; however, a large barrier separates the two regions (Δ*G* = +16 kcal/mol). The latter indicates that diffusion of IP6 from pentamers follows two distinct pathways: IP6 bound above R18 can diffuse toward the exterior of the capsid, while IP6 bound below toward the interior. Together, the PMFs reveal that diffusion of IP6 is primarily driven toward the interior of the capsid.

### Characterization of CypA-DsRed binding to native cores

While the small size of CypA (18 kDa) prohibits reliable three-dimensional (3D) classification of CypA/capsid interaction modes, tetrameric fusion protein CypA-DsRed, where CypA is fused to the fluorescence protein DsRed, provides an adequate molecular mass (186 kDa) for cryoET structural analysis of CypA and capsid interaction modes. In addition, CypA-DsRed has been commonly used as an efficient capsid label for live-cell imaging assays (*30*); structural analysis of its interaction with capsid would provide an understanding of how the probe works. To this end, recombinant CypA-DsRed was purified in a tetrameric form with an estimated molecular mass of 201 kDa (fig. S3A). Two-dimensional class averages of negatively stained CypA-DsRed (fig. S3B) revealed a tetrameric DsRed core, but CypA densities were scattered around it (fig. S3C). Further single-particle cryoEM analysis of CypA-DsRed revealed a well-aligned DsRed tetramer (fig. SD). However, the locations of CypA molecules relative to DsRed are variable, with one, two, or three CypA densities visible in 2D classes (fig. S3D), which impedes structure determination of CypA-DsRed by single-particle cryoEM.

**Fig. 6.**
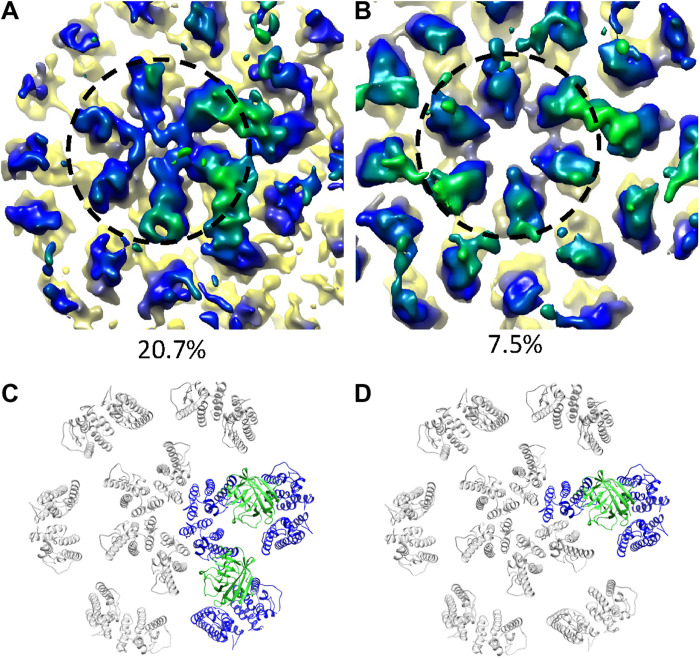
Subtomogram classification and averaging of CypA-DsRed tetramer binding to the native capsid. (**A** and **B**) Density maps of two classes from principal components analysis PCA–based localized classification of capsid/CypA-DsRed complex, with two CypA densities (A) and one CypA density (B). Dashed circle encloses a CA hexamer. (**C** and **D**) Molecular models of the above binding modes, respectively, by rigid-body fitting. CypA is represented by green ribbons, and CypA-interacting CA molecules are represented by blue ribbons.

CypA-DsRed efficiently binds in vitro–assembled WT CA and CA-NC tubes (fig. S4A), but these WT CA tubes were largely bundled, thus not suitable for structural analysis (fig. S4, B and C). A CA mutant, A92E, has been previously used for cryoEM structure determination of CA tubular assemblies because it forms dispersed tubes (*21*). However, CypA-DsRed binding to the CA A92E tubes led to bundling of initially dispersed tubes (fig. S4, D and E). It was apparent that multiple CypA modules within a tetrameric CypA-DsRed can simultaneously bind different tubes and form tube bundles and aggregates. The same effect on tubes was also observed for oligomeric Trim5α and TrimCyp. The capsid in PFO-treated VLPs, however, is protected from coming together by the membrane, making them ideal for studying capsid and CypA-DsRed interaction. We analyzed the structure of PFO-treated HIV-1 cores in complex with CypA-DsRed using subtomogram classification as implemented in emClarity (*35*). Given that four CypA molecules are present within a tetrameric CypA-DsRed molecule, we performed subtomogram classification without imposing any symmetry. The resulting major class (20.7%) showed two CypA modules bound to the capsid, above the CA_CTD_ dimer interface, bridging the CA_NTD_ from neighboring hexamers (Fig. 6, A and C). The second class (7.5%) displayed mainly a single CypA density, while a minor density occupied a pseudo twofold symmetry-related position (Fig. 6, B and D). The remaining classes did not yield any meaningful structure. On the basis of our recent structure of the CypA and CA tube complex, where only one CypA was observed bound above the dimer interface at 33 μM concentration, it is likely that at 12.5 μM concentration, the two bound CypAs observed are from the same CypA-DsRed tetramer. This indicates that the higher affinity of CypA-DsRed to capsid than CypA (*30*) is probably contributed by the multivalent binding of CypA-DsRed. We did not observe densities for DsRed in the maps, understandably, because of the flexible linkage between CypA and DsRed, consistent with the 2D classification results with purified CypA-DsRed (fig. S3, C and D).

## DISCUSSION

IP6 plays an essential role in both the immature and mature HIV-1 capsids, albeit via different mechanisms (*7*, *8*, *27*, *28*). The immature Gag packages IP6 using two rings of lysine residues K158 and K227, at the beginning of a six-helix bundle made of C terminus of CA and space peptide 1, stabilizing the immature Gag lattice (*7*, *39*, *40*). In mature capsid, IP6 is coordinated by the R18 ring in the central cavity of hexamer and pentamer. Previous crystal structures of cross-linked CA hexamers with IP6 and PMFs of hexamers suggest two IP6 binding sites, the one on the top coordinated by R18 and the one on the bottom coordinated by K25 (*6*, *8*, *27*). We consistently observed densities corresponding to two IP6 molecules localized in the center of hexamers within native cores, whereas only the top density was observed in cores of native membraned virions (Fig. 4) (*24*). The difference in the IP6 occupation in hexamers in these two scenarios might be due to the fact that the IP6 molecules in PFO-treated VLPs are in excess in solution, although the IP6 concentrations in both cases are roughly the same (~1 mM). This difference also indicates that the top IP6 binding site has higher affinity than the bottom site.

The bottom IP6 is coordinated by K25 ring, which is important for mature capsid assembly and reverse transcription (*8*). The finding of the additional binding site in HIV-1 CA hexamer in native cores is consistent with the molecular mechanism of the dNTP translocation previously proposed (*27*). Small molecules, such as IP6 and dNTP, bind to the central hexamer cavity. The additional IP6 or dNTPs may facilitate the passage of dNTP through the hexamer pore from the top binding site to the bottom site. On the other hand, the interaction of two IP6 or other polyanions with hexamer can further stabilize the capsid; the dissociation of these polyanions can provide a more controlled uncoating pathway for the metastable capsid.

Tetrameric CypA-DsRed in complex with capsid revealed that two CypA modules can simultaneously bind the capsid (Fig. 6). This multivalent interaction greatly improves the binding capacity of CypA-DsRed to the core compared with monomeric CypA, in agreement with in vitro biophysical measurements (*26*, *30*). The CypA domain in CypA-DsRed bridges two hexamers above the CA_CTD_ dimer interface in a way similar to monomeric CypA binding to CA tubes (*18*, *19*), whereby the underlying CA hexamer structure is essentially undisturbed. This consistency demonstrates that multimerization of CypA, which substantially increases its binding avidity, is a sensible capsid-labeling tool for in situ studies of capsid trafficking, nuclear transport, and uncoating.

Technically, this study established PFO-perforated VLPs as an excellent ex vivo system to study interactions between host cell factors and authentic HIV-1 capsid, circumventing the difficulties inherent with purifying the metastable core from virions. Perforated VLPs enable host cell factors and small molecules to access the native capsid while protecting virus cores. We demonstrated this with high-resolution structures of native HIV-1 capsid in apo form and in the presence of IP6 and CypA or CypA-DsRed by cryoET STA, together with a realistic atomic model of a complete native capsid, which serves as a blueprint for the development of capsid-targeting antivirals. The PFO perforation on enveloped virus membrane provides a novel approach to study host-virus interaction for other viral systems.

## MATERIALS AND METHODS

### HIV-1 *Env*^−^ VLP production and purification

HIV-1 *Env^−^* VLPs were produced by transfecting human embryonic kidney (HEK) 293T Lenti-X cells (catalog no. 632180, Takara/Clontech) with psPAX2 using polyethylenimine (PEI Max) reagent. Two days after transfection, the medium containing the VLPs were harvested and filtered through a 0.45-μm filter. The resulting medium was loaded to an ultracentrifuge (sw 32 rotor) with 3 ml of 8% OptiPrep in STE (saline tris-EDTA) as cushion and centrifuged at 29,000 rpm for 1.5 hours at 4°C. The VLP pellet was collected and loaded to an OptiPrep gradient containing 1 ml of 10, 20, and 30% OptiPrep in STE from the top to bottom. The ultracentrifugation was performed at 45,000 rpm (sw 55 rotor) for 2.5 hours at 4°C. The band corresponding to HIV-1 VLPs was visualized with the help of a light-emitting diode flashlight and collected by puncturing the ultracentrifugation tube in the position where the VLP band is located. The resulting VLP solution was further centrifuged at 42,000 rpm (sw 55 rotor) for 1 hour at 4°C. The supernatant was decanted, and buffer residues were removed by swiping the inner surface of the ultracentrifugation tube with lint-free paper. The final pellet was gently resuspended in 40 μl of STE buffer. The VLP prep was stored at 4°C and used for subsequent studies within 1 day.

### CypA-DsRed expression and purification

The complementary DNA of CypA-DsRED-Express2 [a variant of DsRED (*41*)] was cloned into the pCDNA3.1 mammalian expression vector (Invitrogen Inc.) with the N-terminal Strep affinity tag. HEK293F cells were transiently transfected with the CypA-DsRED expression vector using transfection grade polyethylenimine, molecular weight 25,000 (PEI 25K) (*42*). Cells were harvested 4 days after transfection and lysed with a buffer containing 100 mM tris-HCl (pH 8.0), 150 mM NaCl, 2 mM dithiothreitol (DTT), 0.05% Tween 20, 0.02% sodium azide, and protease inhibitor cocktail (EMD Biosciences Inc.) by sonication. Cell lysates were cleared by centrifugation at 18,000*g*, and Strep-tagged CypA-DsRed was purified with a Strep column (GE Healthcare Life Sciences). The purified protein was buffer-exchanged with 25 mM sodium phosphate (pH 7.5), 150 mM NaCl, 10% glycerol, 2 mM DTT, and 0.02% sodium azide, aliquoted, and flash-frozen in liquid nitrogen.

### PFO production and purification

Expression and purification of PFO (Lys^29^ to the C-terminal end) containing an N-terminal hexahistidine tag were carried out as described previously (*43*). The amino acid sequence for the PFO cysteine-less derivative (PFOC459A) was codon-optimized for *Escherichia coli* expression and cloned into pET-15b (Novagen) by GenScript. Protein expressed from this plasmid is referred to as PFO herein. Hexahistidine-tagged, signal peptide–deficient PFO was transformed into *E. coli* Tuner cells for expression (Novagen). The protein was purified on a Ni column and the hexahistidine tag cleaved by enterokinase. The purified protein was stored in 10% (v/v) glycerol with 50 μM tris(2-carboxyethyl)phosphine (TCEP).

### Binding of CypA-DsRed to CA tube

HIV-1 CA tube assembly (80 μM) was prepared by diluting purified WT CA (10 mg/ml) to 2 mg/ml in a buffer containing 1 M NaCl and 50 mM tris (pH8.0) and incubating at 37°C for 1 hour. The preassembled CA tube was then mixed with CypA-DsRed (6 μM) in the same buffer and incubated at 37°C for 1 hour. The same amount of the CypA-DsRed stock buffer was added for the control samples. At the end of incubation, samples were centrifuged, and the resulting supernatant and pellet were used for SDS–polyacrylamide gel electrophoresis analysis.

### Transmission EM analysis

The morphologies of different variants of CA assemblies and CA/CypA-DsRed complexes were characterized by transmission EM (TEM). Samples were stained with fresh 2% uranyl formate, deposited onto 400-mesh carbon-coated copper grids, and dried for 30 min. TEM images were acquired on a Tecnai T12 transmission electron microscope at 120 kV.

### CryoEM single-particle analysis of CypA-DsRed

For single-particle experiments, purified CypA-DsRed complexes were frozen on graphene oxide-*n*-dodecyl β-d-maltoside (GrOx-DDM) support by plunging into liquid ethane using Vitrobot. GrOx-DDM–coated grids were prepared as described (*44*) with the following difference: Instead of glow discharge, Quantifoil R2/2 grids were washed in 0.006% (w/v) DDM solution before GrOx-DDM mixture application. Data were collected on a Titan Krios electron microscope at 300-keV acceleration voltage and calibrated magnification ×47,170 at the detector level. Images were recorded in counted mode on a K2 direct electron detector mounted after Gatan energy filter. An energy filter slit width of 20 eV was used for data collection. Volta phase plate (*45*) was used to enhance the contrast. Data were recorded in underfocus range 300 to 800 nm and phase shift range π/9 to π/2. Movies of 40 frames were recorded and aligned using MotionCor2 (*46*). Subsequent data analysis was performed in Relion (*47*). Initially, 100,000 particles were selected from ~2000 micrographs. After initial 2D classification, ~60,000 particles far enough from the neighboring particles were selected and reclassified to allow inambiguous identification of CypA densities. Although raw images of the complex suggest the presence of multiple copies of CypA per DsRed tetramer, many classes show only one to three CypA densities (fig. S3) because of the flexibility of the linker, which might result in CypA densities being averaged out.

### CryoET sample preparation

PFO concentration was titrated against the VLP sample to obtain an optimal pore formation on the VLP membrane by negative-staining EM. One microliter of diluted PFO was added to 10 μl of VLP stock and incubated at room temperature for 30 min before plunge-freezing. For VLPs in complex with IP6/CypA or IP6/CypA-DsRed, IP6 was added to PFO-perforated VLPs at a final concentration of 1 mM before incubation with 25 μM CypA or 12.5 μM CypA-DsRed at 25°C for 30 min. The VLPs were mixed with 6-nm gold beads before plunge-freezing onto glow-discharged Quantifoil grids (300-mesh R2/2) or Lacey carbon-coated copper grids (300 mesh, Agar Scientific), with Leica GP2 or Vitrobot. Three microliters of the sample was added onto the carbon side of the grid and 1 μl onto the mesh side, and then the grid was blotted from the mesh side for 3 s before plunge-freezing into liquid ethane. The humidity was set to 100% during blotting.

### CryoET data collection

Cryo-electron tilt series were acquired using Thermo Fisher Scientific Titan Krios operated at 300 keV equipped with a Gatan Quantum postcolumn energy filter operated in zero-loss mode with 20 eV slit width and a Gatan K2/K3 direct electron detector in eBIC (electron Bio-Imaging Centre, Diamond). Tilt series were collected with SerialEM (*48*) with a nominal magnification of 130k/81k. They were acquired using a dose-symmetric tilt scheme (*49*) starting from 0° with a 3° tilt increment by a group of three and an angular range of ±60°. The accumulated dose of each tilt series was around 120 e^−^/Å^2^ with a defocus range between −1.5 and −7 μm. Ten frames were saved in each raw tilt stack. Details of data collection parameters are listed in Table 1.

### CryoET data processing and STA/subtomogram classification

An in-house script for the preprocessing of the raw frames was used (https://github.com/ffyr2w/cet_toolbox) to perform motion correction (*46*) of the raw frames and tilt series alignment using gold fiducial beads in eTOMO batchmode (*50*). The fiducial markers were manually inspected to ensure the centering of predicted markers for each tilt series.

STA was performed following the workflow of emClarity (*35*). A seven-hexamer unit density was cropped from a helical reconstruction of CA lattice and used as the template to find the initial positions of hexamers in the cone. The template was band-pass–filtered to around ~25 Å, and the template matching was carried out with 8× binned tomograms with a pixel size of ~8.5 Å. The false-positive positions of hexamers that do not satisfy the hexagonal lattice geometry were removed automatically, with further manual inspections. The hexamers were then aligned together iteratively using 6×, 4×, 3×, and 2× binned tomograms, with three rounds of tomoCPR between transition from 6× to 4×, from 4× to 3× and from 3× to 2× binned tomograms, respectively. A cylindrical alignment mask enclosing seven hexamers and sixfold symmetry were used throughout the alignment procedure. The final density map was reconstructed at bin 1 using cisTEM within emClarity package (emClarity v1.5.1.04), in which the raw particle projections corresponding to the subtomograms were reextracted from raw tilt series and reconstructed using the Fourier reconstruction approach implemented in cisTEM. The final reconstructed maps were sharpened with a B-factor of −100. To test whether the central density in the hexamer is an artifact due to the sixfold symmetrization during STA, we performed subtomogram alignment and averaging without symmetry throughout the pipeline and generated the final reconstruction through cisTEM in emClarity package (fig. S2).

The pentamers on the capsid were searched by template matching in a manner similar to the procedure performed for hexamer. The high cross-correlation peaks corresponding to true positive pentamers were identified manually, guided by the existing hexamer lattice obtained from the hexamer template search. The tomograms were iteratively aligned and averaged with a fivefold symmetry to obtain the final density map, without tomoCPR between transitions among different binnings. The final map was reconstructed at bin1 and sharpened with a B-factor of −10 (Table 1).

The classification of CypA-DsRed–bound CA hexamer was performed in a 2× binned tomogram. The hexamers were aligned in 2× binned sirt-filtered tomograms for several iterations to relax its symmetry form C6 to C1. Principal components analysis–based conformational classification in emClarity was performed with a local mask on the central hexamer and classified subtomograms to 16 classes. Three classes with 10,295 subtomograms showed two neighboring CypA densities. One class with 3,734 subtomograms showed mainly one CypA density. For two-CypA–binding classes, we rotated the subtomograms to one direction on the basis of the CypA positions and combined them together for further average and alignment. The further average and alignment for both of the two conformational states were performed in 2× and 1× binned original tomograms.

### Model building and refinement and analysis

The CA hexamers (PDB 4XFX) were used as the initial model to rigid-body fit into the density map in Chimera (*51*), with CA_NTD_ and CA_CTD_ as two rigid bodies and manually improved the mainchain in Coot (*52*). The initial model for CypA was based on the crystal structure (PDB 1AK4). Two IP6 molecules were derived from crystal structure (PDB 6BHT) and modeled (fig. 4) by aligning the CA hexamers from our density maps to the cross-linked CA hexamer in 6BHT. We noticed a slight difference in the microscope pixel size between the data of membrane-enclosed HIV-1 capsid (EMD-3465) and our data and rescaled the hexamer map (EMD-3465) to match our hexamer map in the apo form. The rescaled hexamer map with pixel size 1.712 Å was used as a reference to generate a new hexamer model for comparison. Structure alignment and comparison were conducted using *matchmaker* in Chimera (*51*).

### Preparation of cryoET-derived CA hexamer and pentamer for MD simulation

To obtain an MD-ready model of the CA hexamer and pentamer, flexible fitting of the atomic structure into the cryoEM density was performed using the molecular dynamics flexible fitting (MDFF) method as follows (*53*). First, missing loops were added to the models using MODELLER (*54*). Subsequently, ions near the surface of the protein were placed using Cionize (*55*). Protonation states of titratable residues were assigned using PDB 2PQR (*56*) at pH 7.4. Bulk water and Na/Cl ions were then added using visual molecular dynamics (VMD), setting the total concentration of NaCl to 150 mM, resulting in 100,000 total atoms, including solvent. The models were solvated using the TIP3P (transferable intermolecular potential with 3 points) water model (*57*). The systems were then thermalized at 310 K and 1 atm for 5 ns while applying positional restraints on all heavy atoms of the protein. MDFF was then applied for 10 ns with a grid scaling of φ = 0.05 coupled only to backbone heavy atoms. Domain restraints were applied to maintain the structural integrity of each CA domain. To maintain secondary structure integrity and to prevent transitions of cis/trans bonds and chirality errors, additional restraints were used in the potential (*58*). MDFF was performed using NAMD 2.14 (*59*) with the CHARMM36m force field.

### Molecular mechanics parameterization of IP6

Parameters for IP6 were derived by analogy following the CHARMM General Force fFeld (CGENFF) protocol (*60*). The parameter penalties and charge penalties in each generated parameter files were less than 10, indicating good analogy with the available atom types present in CGENFF.

### Simulation setup for IP6-bound CA hexamer and pentamer

The small molecule IP6 was placed in the central cavity of the thermalized/equilibrated CA hexamer/pentamer models. The length of the system in the ẑ direction provided sufficient solvent padding, greater than 24 Å, to avoid interactions between periodic images. Na and Cl ions were then added to neutralize the system using the CIONIZE plugin in VMD (*61*), and the bulk salt concentration was set to the physiological concentration of 150 mM, as previously described by the authors (*27*, *62*). The total number of atoms of the resulting CA hexamer/pentamer models was 100,000.

### System minimization and equilibration

Solvated hexamers and pentamers were subjected to minimization using the conjugated gradient algorithm with linear searching as implemented in NAMD (*55*). During minimization, the first 10,000 minimization were applied where only water molecules and ions were free to move while the CA protein and IP6 were fixed. Subsequently, the backbone atoms of the CA protein were mobile but restrained with a force constant of 10.0 kcal mol^−1^ Å^−2^. Convergence of the minimization procedure was evaluated until the variance of the energy gradient was less than 0.1 kcal mol^−1^ Å^−1^. After minimization, the systems were thermalized from 0 to 310 K in increments of 20 K over 1 ns.

Equilibration of the systems was achieved at 310 K during 100,000 MD steps, while the protein backbone atoms were restrained, and convergence of equilibration was determined by monitoring the evolution of the RMSD over time, which converged at 2 Å. Positional restraints were gradually released at a rate of 1.0 Kcal mol^−1^ Å^−2^ per 400 ps from 10.0 to 0.0 Kcal mol^−1^ Å^−2^. All MD simulations were performed using NAMD 2.14 with the CHARMM36m (*63*) force field with a cutoff for short-range electrostatics interactions of 12 Å (*55*, *64*, *65*). An internal time step of 2 fs was used in the multistep reversible reference system propagation algorithm (r-RESPA) integrator as implemented in NAMD, bonded interactions were evaluated every 2 fs, and electrostatics were updated every 4 fs. Temperature was held constant at 310 K using a stochastic rescaling thermostat. Pressure was controlled at 1 bar using a Nose-Hoover Langevin piston barostat with period and decay of 40 and 10 ps, respectively. Long-range electrostatics was calculated using the particle mesh Ewald summation with a grid size of 1 Å.

### Gibbs free energy calculations

For all free-energy MD calculations, a reaction coordinate was defined as the location of the center of mass of IP6 along the *Z* axis. The origin of the reaction coordinate was set to the center of mass of C_α_ atoms in the N-terminal domain of either a CA hexamer or pentamer; note that because of the structural differences between pentamers and hexamers, the reaction coordinate is incongruent. One-dimensional PMFs along the reaction coordinate were calculated using the Hamiltonian Replica-exchange/Umbrella Sampling (HREX/US) method (*27*, *66*–*68*) implemented in NAMD 2.14 (*55*, *66*, *69*). The initial coordinates for the HREX/US windows were derived from constant-velocity steered MD simulations where IP6 was displaced along the progress variable (PV) at a rate of 0.1 nm/ns; last, all HREX simulations were separated with an interreplica distance of 0.5 Å. The center of mass of IP6 was positionally restrained within simulation windows using a harmonic force constant of 2.5 kcal mol^−1^ Å^−2^, the latter implemented in NAMD by the Colvars (*67*) extension.

During HREX/US simulations, exchange of the harmonic potential between neighboring replicas was attempted every 1000 steps (2 ps); these exchanges were evaluated between successive simulation windows. Exchange attempts between any given simulation window was equally probable. To determine the exchange probability between simulation windows, the following Metropolis Monte Carlo exchange criterion (*68*) was used

$$p_{\text{exchange}}=\text{min}(1,e^{-\frac{\left[U_{i}(q_{i})-U_{i}(q_{j})]+[U_{j}(q_{j})-U_{j}(q_{i})\right]}{k_{B}T}})$$

where *T* = 310 K, *k*_B_ is the Boltzmann constant, *q_i_* and *q_j_* denote the 3D conformations for two replicas *i* and *j*, and *U_i_* and *U_j_* represent the potential energies derived from the restrained Hamiltonian evaluated at the indicated conformation.

PMF were derived from the resulting sampling in each of the simulation windows using the weighed histogram analysis method (WHAM) (*70*, *71*). In WHAM, PMF-based bins are obtained from US/HREX simulation windows. Convergence of the 1D US/HREX calculations was characterized by the changes in the resulting PMF in trajectory increments of 10 ns. That is, simulations were considered to have converged once the maximum change in one of the PMF bins, resulting from adding more simulation data, was less than 1 kcal mol^−1^.

### Model building of a fullerenic HIV-1 capsid model

The fullerenic model of the HIV-1 capsid composed of 241 CA hexamers and 12 CA pentamers, with the ring spiral pentagon indices 1, 2, 9, 15, 17, 198, 203, 207, 212, 217, 230, and 239 (Fig. 2) (*21*), was embedded in a water box with 500 mM NaCl, resulting in a simulation box of dimensions 75 nm by 75 nm by 135 nm and a total of 76,000,000 atoms, as described in a previous publication (*17*). In particular, the sequence of HIV-1 subtype B, NL4-3, was used for all CA monomers. For the present study, MD simulations of the unrestrained, fully solvated HIV-1 empty capsid were performed using NAMD 2.14 (*55*) for a total of 10 ns. The CHARMM36m (*63*) force field was used with the TIP3P (*57*) water model at 310 K and 1 atm. Simulations carried out in the present study used the r-RESPA integrator available in NAMD. Long-range electrostatic force calculations used the particle mesh Ewald method using a grid spacing of 2.1 Å and eighth-order interpolation, with a 1.2-nm cutoff. The simulations used an integration time step of 2 fs, with nonbonded interactions evaluated every 2 fs and electrostatics updated every 4 fs; all hydrogen bonds were constrained with the SHAKE algorithm.

## Notes

View/request a protocol for this paper from *Bio-protocol*.
