# Supplementary material for: Structure of native HIV-1 cores and their interactions with IP6 and CypA
Source: Sci Adv. 2021 Nov 19;7(47):eabj5715. doi: 10.1126/sciadv.abj5715 (PMC8604400; doi:10.1126/sciadv.abj5715)
Supplement: Supplementary file 1 — Table S1 Figs. S1 to S4 [file sciadv.abj5715_sm.pdf]

Science Advances
AAAS

Supplementary Materials for

Structure of native HIV-1 cores and their interactions with IP6 and CypA

Tao Ni, Yanan Zhu, Zhengyi Yang, Chaoyi Xu, Yuriy Chaban, Tanya Nesterova, Jiying Ning, Till Böcking, Michael W. Parker, Christina Monnie, Jinwoo Ahn, Juan R. Perilla, Peijun Zhang*

*Corresponding author. Email: peijun.zhang@strubi.ox.ac.uk

Published 19 November 2021, *Sci. Adv.* **7**, eabj5715 (2021)
DOI: 10.1126/sciadv.abj5715

This PDF file includes:

Table S1
Figs. S1 to S4

Table S1. Comparison of subtomogram averaging using PFO-treated virions and those without treatment(Mattei et al. (ref 24)).

	# of tilt series	# of cores	# of subtomograms	Pixel size (Å)	Defocus (µm)	Resolution (Å)
**Cores Mattei et al**	103	552	72,836	1.78	-2 to -6.5	6.8
**PFO-Cores This study**	101	157	32,114	1.04	-2 to -3	5.8
**PFO-Cores + IP6 This study**	109	481	82,837	1.06	-2.5 to -7	5.4

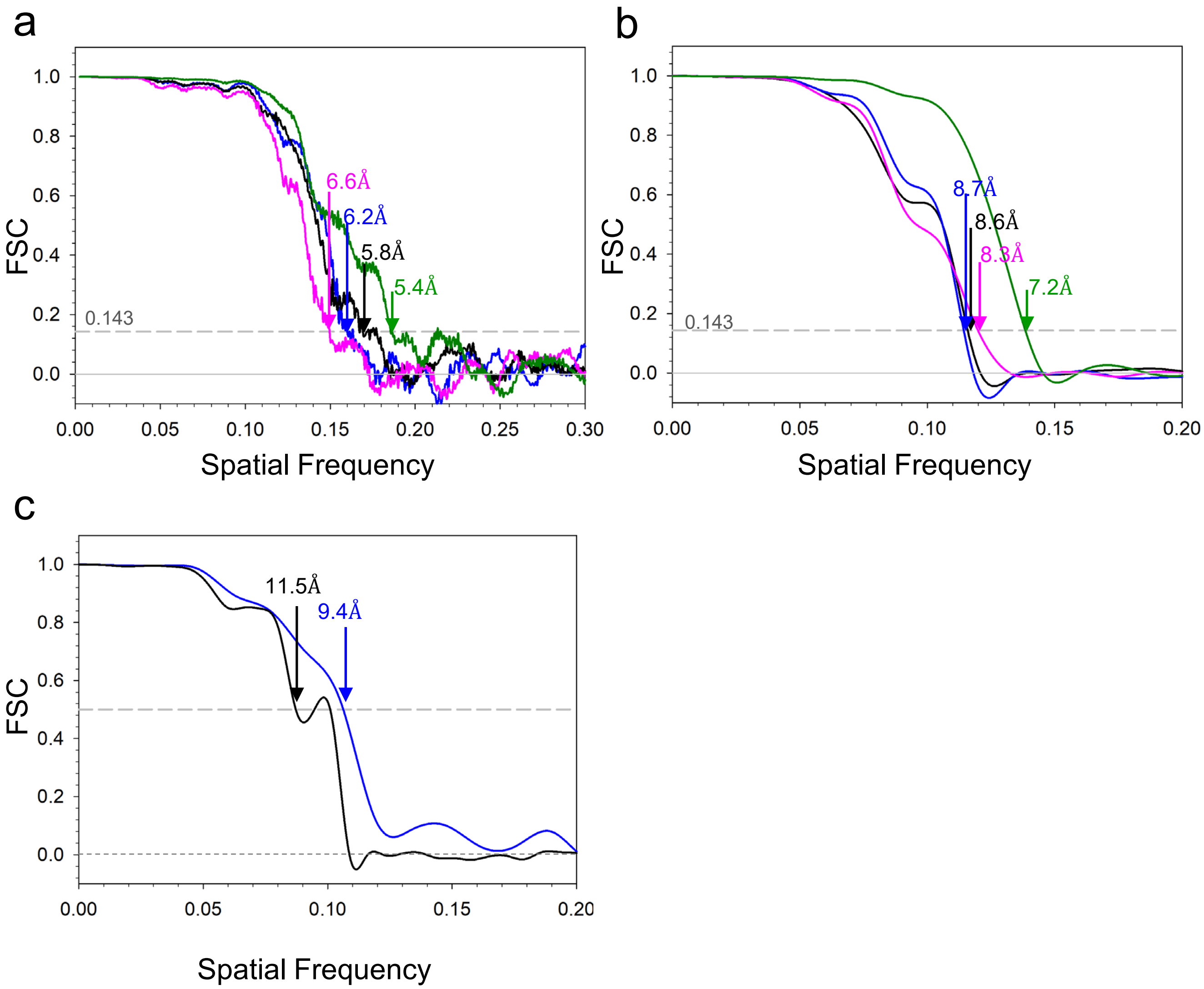

Figure S1 | Fourier Shell Correlations (FSC) of HIV-1 capsid capsomer maps. a) FSC plots of CA hexamers in the apo state (black), in the presence of IP6/CypA (blue), IP6/CypA-DsRed (pink). b) FSC plots of CA pentamers in the apo state (black), in the presence of IP6/CypA (blue) and IP6/CypA-DsRed (pink). c) FSC plots of CypA-DsRed in complex with CA hexamers from subtomogram classes of one CypA-DsRed (black) and two CypA-DsRed (blue) bound to CA hexamers. A soft mask enclosing the central hexamer was used during FSCs calculation.

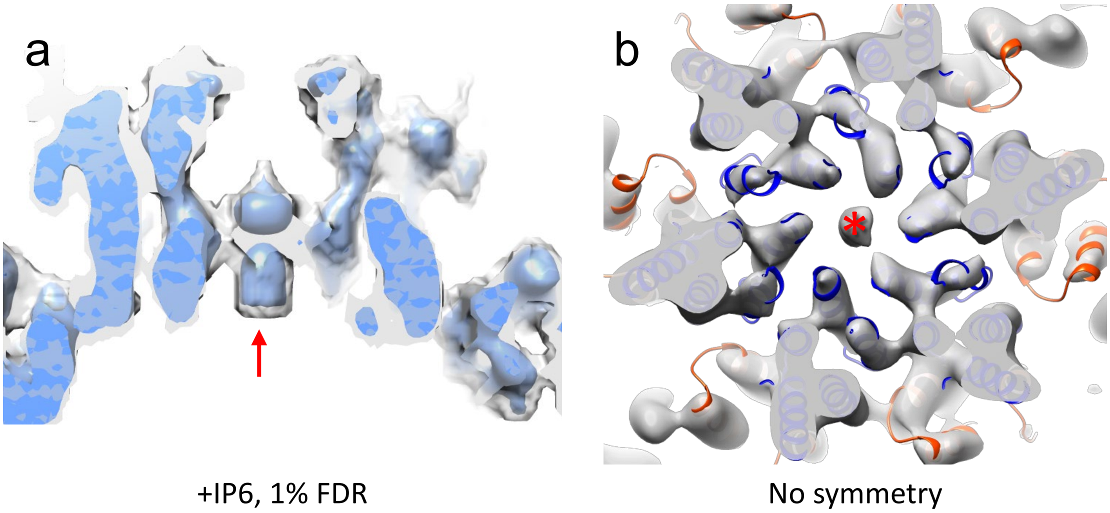

Figure S2 | Analysis of the central density in IP6/CA hexamer and IP6/CypA-DsRed/CA hexamer complexes. a) Side view of confidence map at 1% false discovery rate (transparent gray) overlaid with IP6/CA hexamer map (blue). b) Subtomogram averaging of IP6/CypA-DsRed/CA hexamer without applying six-fold symmetry throughout the processing. The red asterisk and arrow indicate the central density.

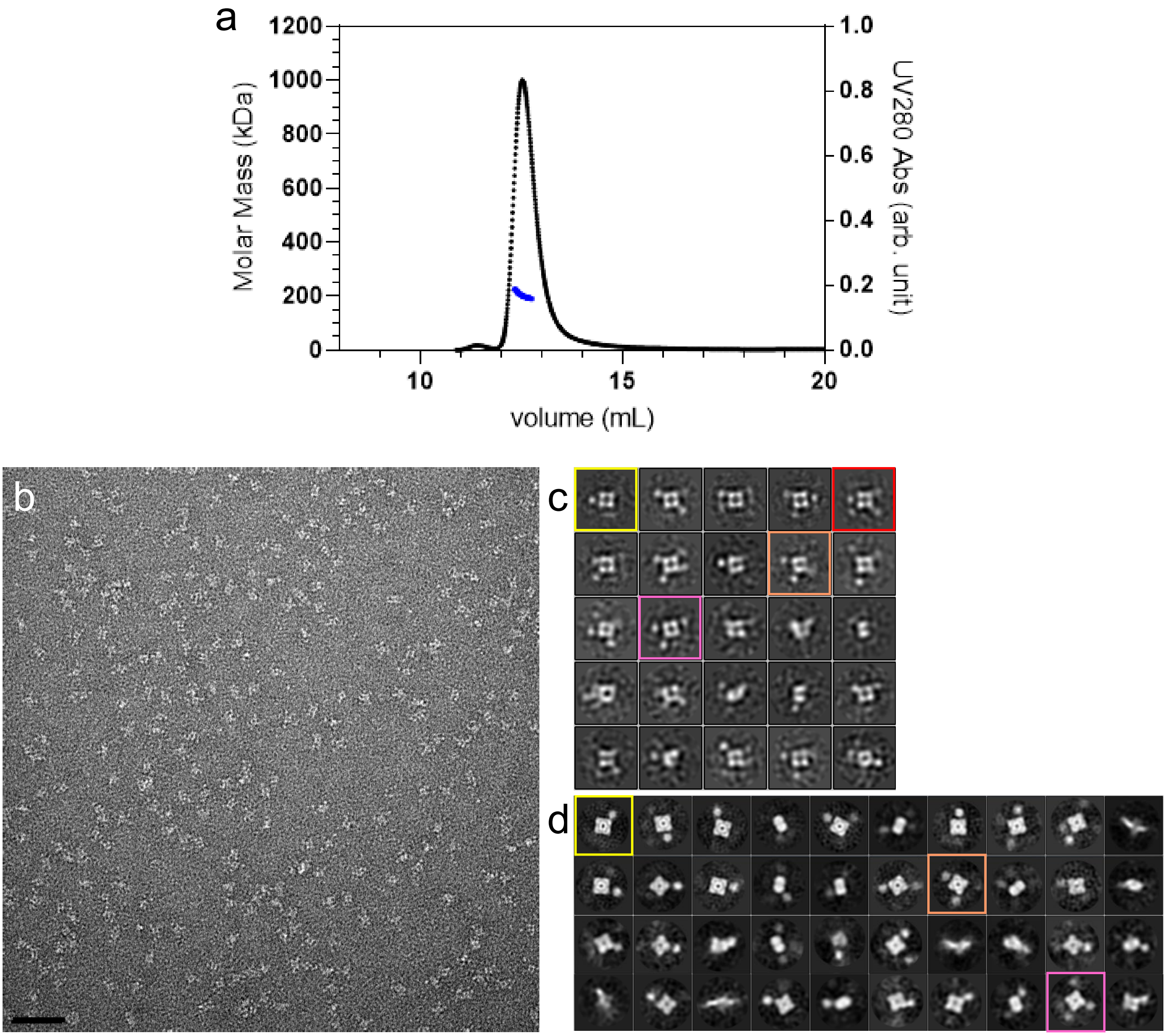

Figure S3 | Purification and characterization of CypA-DsRed. a) Multi-angle light scattering of CypA-DsRed. The protein at 3.0 mg/mL was injected into an analytical Superdex 200 gel filtration column at a flow rate of 0.5 ml/min. Molecular mass of CypA-DsRed (UV absorbance at 280 nm in black solid circle) at the peak elution is indicated with blue solid circle. The estimated average molecular mass of CypA-DsRed across the peak is 201 kDa. b) A negative stain image of purified CypA-DsRed. c-d) 2D classes of CypA-DsRed particles from negative stained images (c) and cryoEM images recorded with a phase plate (d). Yellow, orange, pink and red boxed classes display representative one, two, three and four CypA densities respectively in CypA-DsRed tetramer particles. Scale bar, 50 nm

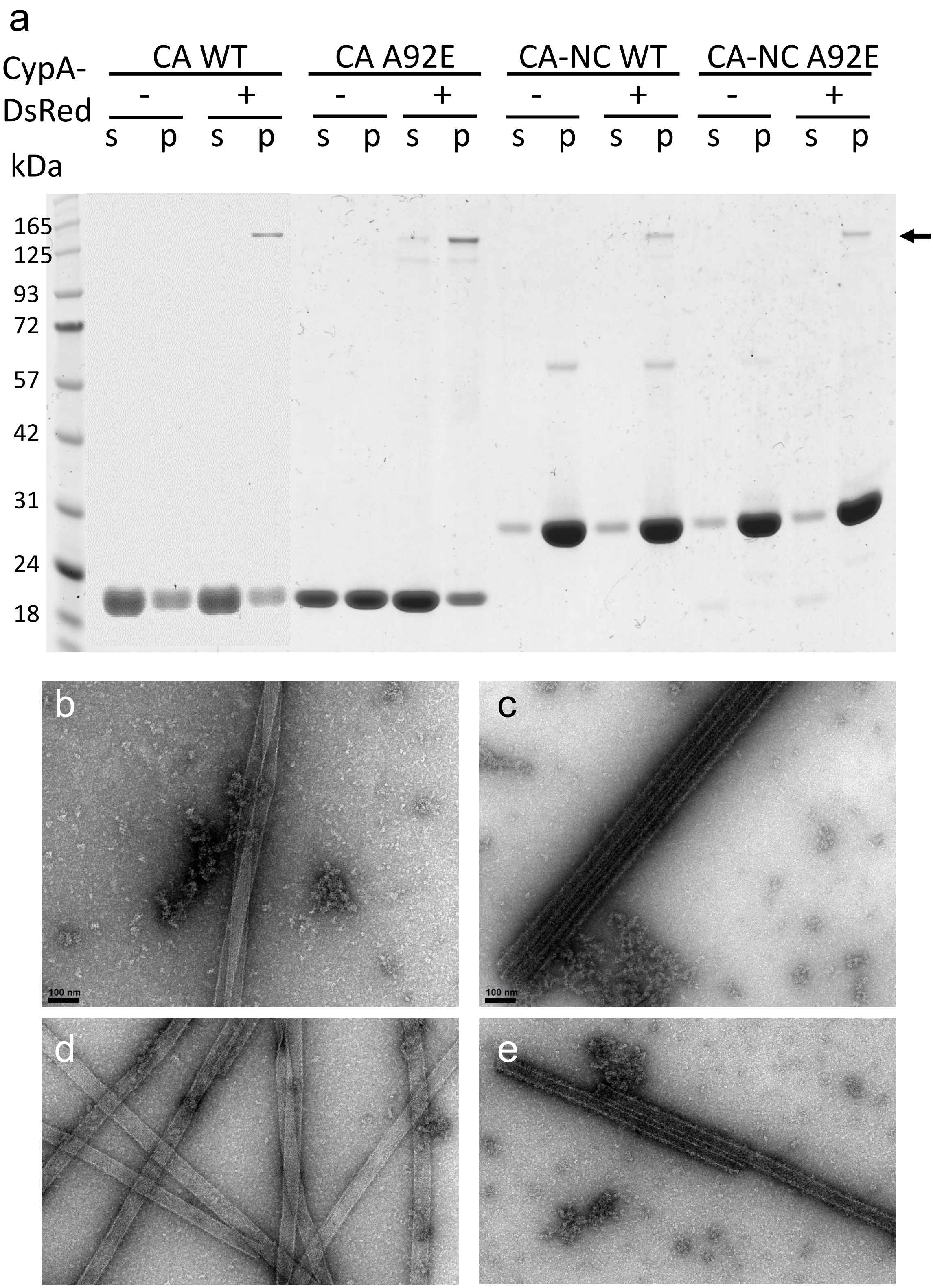

Figure S4 | Binding of CypA-DsRed to capsid assemblies. a) SDS-PAGE analysis of CypA-DsRed binding to WT and A92E CA tubes and CA-NC tubes following pelleting assay, stained with Coomassie Blue. CypA-DsRed runs as a tetramer, marked by an arrow. b-e) Negative stain images of reaction mix from (a) for WT CA assemblies in the absence (b) and presence (c) of CypA-DsRed. d-e) Negative stain images of reaction mix from (a) for A92E CA assemblies in the absence (d) and presence (e) of CypA-DsRed. Scale bars, 100 nm
